# Hepatitis C in the era of direct-acting antivirals: real-world costs of untreated chronic hepatitis C; a cross-sectional study

**DOI:** 10.1186/s12879-015-1208-1

**Published:** 2015-10-26

**Authors:** Jennifer Ann Kieran, Suzanne Norris, Aisling O’Leary, Cathal Walsh, Raphael Merriman, D. Houlihan, P. Aiden McCormick, Susan McKiernan, Colm Bergin, Michael Barry

**Affiliations:** Department of Pharmacology and Therapeutics, Trinity College Dublin, Dublin, Ireland; National Centre for Pharmacoeconomics, St. James Hospital, Dublin, Ireland; Department of Hepatology, St. James Hospital, Dublin, Ireland; MACSI and Health Research Institute, University of Limerick, Limerick, Ireland; Liver Unit, St. Vincent’s University Hospital, Dublin, Ireland; Department of Genitourinary Medicine and Infectious Diseases, St. James Hospital, Dublin, Ireland; School of Medicine, Trinity College Dublin, Dublin, Ireland; School of Pharmacy, Royal College of Surgeons of Ireland, Dublin, Ireland

**Keywords:** Ireland, Health economics, Direct medical cost, Hepatitis C, Pharmacoeconomics

## Abstract

**Background:**

Recent advances in Hepatitis C therapeutics offer the possibility of cure but will be expensive. The cost of treatment may be partially offset by the avoidance of advanced liver disease. We performed a micro-costing study of the ambulatory healthcare utilisation of patients with Hepatitis C supplemented with inpatient diagnosis related group costs.

**Methods:**

The staff utilisation costs associated with a Hepatitis C ambulatory visit were measured and combined with the costs of investigations to establish a mean cost per consultation. An annualised estimate of cost was produced by multiplying this by the number of consultations accessed, stratified by degree of liver impairment. Inpatient costs were established by identifying the number of inpatient episodes and multiplying by Irish diagnosis related group costs. Non-parametric bootstrapping was performed to derive mean and 95%CI values.

**Results:**

Two hundred and twenty-five patients were identified. The cost of an outpatient medical review was €136 (€3.60 SD). The cost of a Hepatitis C nursing review was €128 (€7.30 SD). The annual mean costs of care were as follows (95%CI): Mild €398 (€336, €482), Moderate €417(€335, €503), Compensated cirrhosis €1790 (€990, €3164), Decompensated cirrhosis €8302 (€3945, €14,637), Transplantation Year 1 €137,176 (€136,024, €138,306), Transplantation after Year 1 €5337 (€4942, €5799), Hepatocellular carcinoma €21,992 (€15,222, €29,467), Sustained virological response €44 (€16, €73).

**Conclusions:**

The direct medical cost associated with Hepatitis C care in Ireland is substantial and increases exponentially with progression of liver disease. The follow-up costs of patients with a sustained virological response in this cohort were low in comparison to patients with chronic infection.

**Electronic supplementary material:**

The online version of this article (doi:10.1186/s12879-015-1208-1) contains supplementary material, which is available to authorized users.

## Background

Chronic Hepatitis C (HCV) is an important public health challenge with the World Health Organisation estimating that 185 million people are infected worldwide [1]. There is an estimated 20,000 to 50,000 people chronically infected with HCV in Ireland, the majority of whom are yet to be diagnosed [2]. In its later stages, HCV causes significant morbidity and mortality. Given the clinical and economic burden caused by end-stage liver disease from HCV, there is a drive in many healthcare systems to diagnose and treat people with HCV before they develop clinical disease [3, 4]. After a decade of little development in HCV therapy, there is presently a rapidly expanding therapeutic armamentarium. Currently licensed direct acting anti-virals (DAA’s) significantly improve the treatment outcomes for patients with HCV genotype 1 infection and further novel agents promise a paradigm shift in the treatments and outcomes for patients with HCV of all genotypes [3, 5–10]. These novel agents demonstrate excellent clinical efficacy but are more costly than the previous standard of care, pegylated interferon and ribavirin, which has been shown to have a substantial budget impact in Ireland [11]. The budget impact of the DAA’s make their affordability challenging in many healthcare systems, even when they are found to be cost-effective. In order to accurately define the cost-effectiveness of these agents in the Irish healthcare setting, a research project was undertaken to define the direct medical costs of HCV care in Ireland.

## **Methods**

The majority of care provided for patients with HCV is ambulatory in nature with inpatient care required in some patients in the later stages of disease. A bottom-up micro-costing project was undertaken at two large tertiary referral hepatology services (Institution 1 and Institution 2) in Ireland to establish the cost of ambulatory HCV care. In order to give a more complete picture of the cost of HCV care, unit costs of hospital based procedures and admissions were sourced from Irish Diagnosis Related Group (DRG) costs and added to the ambulatory care costs where appropriate. Over 4000 HCV patients are registered for care between these Institutions, representing approximately half of the HCV patients currently diagnosed in Ireland [12]. The sample taken encompasses patients along the full spectrum of HCV-related liver disease and is representative of clinical care in Ireland, which is funded and delivered through the public health service. The cost of care associated with HCV treatment was not included in this study.

### Micro-costing of ambulatory care

In order to establish the staff utilisation involved in providing an outpatient HCV consultation, the time spent by staff in providing consultations was measured. Ninety patients had their medical consultation timed (51 patients from an unselected HCV cohort with all stages of disease severity, 39 patients from the transplant service), 11 patients had their nursing consultation timed, 81 patients had their blood draw timed and 68 patients had their administrative encounter timed. The unit cost associated with this time was calculated using the mid-point of the Health Service Executive (HSE) salary scales for new-entrants in 2010 and adjusted for non-pay salary cost as per guidelines [13–15].

Hospital electronic records were interrogated to establish the ordering frequency of a set of pertinent investigations (Table 1) in a cohort of HCV patients for three years between 2011 and 2013. Sixty-seven patient records were analysed (39 patients with chronic mild HCV infection not undergoing HCV treatment and 28 patients with a sustained virological response (SVR)). The unit costs applied to these investigations were obtained from laboratory and radiology costs supplied by the finance departments at the participating hospitals and the National Viral Reference Laboratory at University College Dublin. The costs of these investigations were divided by the number of outpatient visits attended by the patient to derive a mean cost of investigations per visit. The mean costs derived for staff and laboratory utilisation were combined to develop an overall cost for a HCV outpatient review.

**Table 1:** Health resource utilisation considered in derivation of cost of HCV Health-states

Input	Mild	Mod	Comp. Cirrhosis	Decomp. Cirrhosis	HCC	Transplant Year 1	Transplant > Year 1	SVR	Source
OPD bundle^a^	✓	✓	✓	✓	✓	✓	✓	✓	Institution costs
FBC									
Renal profile									
Liver profile									
Coagulation screen									
AFP									
HCV Viral load									
Liver US									
Staff Costs									HSE salary scale 2010
Liver Biopsy	✓	✓	✓	✕	✕	✕	✕	✕	HSE DRG 2011
OGD^b^	✕	✕	✓	✓	✓	✕	✕	✕	HSE DRG 2011
Hepatology Medical review	✓	✓	✓	✓	✓	✓	✓	✓	Micro-costing
Hepatology Nursing review	✓	✓	✓	✓	✓	✕	✕	✕	Micro-costing
Inpatient Admission	✕	✕	✓	✓	✓	✓	✓	✕	HSE DRG 2011
Sorafenib	✕	✕	✕	✕	✓	✕	✕	✕	Micro-costing^d^
TACE^c^	✕	✕	✕	✕	✓	✕	✕	✕	Institution cost
Oncology/ Palliative OPD	✕	✕	✕	✕	✓	✕	✕	✕	HSE DRG 2011
Dermatology OPD	✕	✕	✕	✕	✕	✓	✓	✕	HSE DRG 2011
Hepatology Dayward review	✕	✕	✕	✕	✕	✓	✓	✕	HSE DRG 2011
Pre-transplant workup	✕	✕	✕	✕	✕	✓	✕	✕	Micro-costing
3 months Prophylaxis	✕	✕	✕	✕	✕	✓	✕	✕	Micro-costing^d^
Immunosuppression	✕	✕	✕	✕	✕	✓	✓	✕	Micro-costing^d^
Diuretic/Beta-blocker	✕	✕	✓	✓	✕	✕	✕	✕	Micro-costing^d^

*Mod* moderate, *Comp. cirrhosis* Compensated cirrhosis, *Decomp. Cirrhosis* decompensated cirrhosis, *HCC* hepatocellular carcinoma, *Transplant Year 1* First 12 months of liver transplantation, *Transplantation > Year 1* Transplantation After First 12 months, *SVR* Sustained Viral Response, *HSE* Health Service Executive, *DRG* Diagnostic Related Group, *PCRS* Patient Care Reimbursement Service, *OPD* outpatient, ^a^Bundle: *FBC* Full Blood Count, *Renal Profile* Urea, Creatinine, Sodium, Potassium, Bicarbonate; *Liver Profile* Albumin, Alanine amino transaminase, Aspartate amino transaminase, Gamma glutamyl transferase, Bilirubin, Lactate Dehydrogenase; *Coagulation Screen* Prothrombin Time, Activated partial thromboplastin time, International Normalised Ratio, *AFP* Alpha fetoprotein, ^b^ *OGD* Oesophago-gastro-duodenoscopy, ^c^ *TACE* Transarterial chemoembolisation, ^d^Unit cost of drug sourced from the Primary Care Reimbursement System

In order to establish the number of clinical reviews accessed by patients with HCV, a cohort of 225 patients with HCV attending the two units was identified and stratified into health-states according to clinical, radiological and histological criteria. These are consistent with the natural history of HCV. The health-states established were: mild, moderate, compensated cirrhosis, liver transplantation Year 1, liver transplantation after Year 1, decompensated cirrhosis, hepatocellular carcinoma and sustained virological response (SVR). The hospital electronic patient records (EPR) of the two units were interrogated to establish how many annual consultations by medical and nursing staff each patient accessed from 2006 to 2012 and this was multiplied by the cost of review established during the micro-costing project. Consultations that took place over the course of HCV treatment were excluded. Patient who had achieved an SVR were assessed for the consultations they accessed post successful treatment from 2011 to 2013 inclusive. This produced an annual cost of ambulatory care for patients with differing levels of HCV disease severity.

### Number and cost of inpatient episodes for end-stage liver disease

In addition to the ambulatory care costs, patients with more severe forms of liver disease such as decompensated cirrhosis, hepatocellular carcinoma and liver transplantation have significant inpatient costs associated with their care. A cohort of 13 patients with decompensated cirrhosis and 27 patients with hepatocellular carcinoma was identified from the prospectively collected clinical database of the Hepatology service of Institution 1. The number of inpatient and outpatient clinical episodes for the last three years of follow up or from diagnosis to death was established through interrogation of the EPR. The HSE DRG cost of an inpatient admission with liver disease from 2011 was applied to the inpatient stays with outpatient costs applied as per the micro-costing study. In addition, the duration of prescriptions in months of high-cost drugs such as sorafenib and the frequency of high-cost procedures such as Trans-arterial chemo-embolisation (TACE) were established and included in the cost estimates for HCC patients. The drug cost of sorafenib was taken from the standard unit costs from the High-tech Drug Scheme in Ireland, adjusted as per guidelines from the National Centre for Pharmacoeconomics in Ireland [14].

### Cost of liver transplantation and post-transplantation care

The cost of liver transplantation in the first year post-transplantation was established through a combination of bottom-up micro-costing of the ambulatory care component supplemented by the DRG cost of the inpatient liver transplantation procedure. The cost of the pre-transplantation work-up was determined by micro-costing the pre-transplantation work-up protocol of Institution 2, which is the sole institution delivering liver transplantation services in the Republic of Ireland. The number of outpatient consultations and inpatient admissions required by 33 patients in the immediate year post-transplantation and in 31 patients in the years thereafter was established from the EPR of Institution 2 and the outpatient cost established from the micro-costing study was applied to outpatient consultations with the DRG cost of an inpatient admission with liver disease in 2011 used for the inpatient admission. The prescription of immunosuppressive therapies post-transplantation was established from the prospectively collected transplantation database in Institution 2 and the costs of these prescriptions was calculated using standard unit costs from the High-tech Drug Scheme in Ireland, adjusted as per guidelines from the National Centre for Pharmacoeconomics in Ireland [14]. The rate of valganciclovir prescription for post-transplantation prophylaxis was estimated at 0.21 based on the rate of cytomegalovirus seroprevalence in a cohort of Irish pregnant women [15]. These drug costs were added to the outpatient and inpatient resources consumed to give a total cost for post-transplantation care.

All costs had annual inflation of 4 % applied as per guidance from the Irish Department of Finance. Table 1 summarises the health resources evaluated and included in each health-state and their source.

### Statistical analysis

Descriptive statistics were performed on the results from the micro-costing project to give average values with 95 % Confidence Intervals (CI). Mean time required for clinical review was examined for significant differences depending on provider and clinical indication using an independent *t*-test for those elements with two categories and a one-way ANOVA for those with greater than two categories. A p-value of less than 0.05 was considered significant. SPSS V21 was used for this analysis.

Given the positive skew of the annual cost estimates for the health-states, non-parametric bootstrapping was performed to establish valid means. Mean and 95 % Confidence intervals (95 % CI) are presented. The analysis was performed using Microsoft Excel.

This study received approval from the Ethics Committee of St James’s Hospital, Dublin and St Vincent’s University Hospital, Dublin.

## Results

### Micro-costing of ambulatory care

#### Medical review

The average time spent per medical review was 17 min. (95 % CI 15,19). Mean review times in both clinical sites were the same. There was a significant difference in the time required for a clinical consultation undertaken by a doctor who had completed speciality training (consultant) compared to one performed by a doctor-in-training (mean time 13 versus 19 min *p* = 0.001) and in the time required to review a patient depending on their indication for review, as those with chronic HCV required more time. Patients attending for a first review required an average of 16 min (95 % CI 13,19), those for routine HCV monitoring required an average of 18 min (95 % CI 16, 20), while those attending for review post-sustained viral response required on average 8 min (95%CI 6,9) *p* = 0.03.

#### Nursing review

Clinical nurse specialist reviews of patients being assessed for HCV treatment were timed. Those for pre-treatment assessment or work-up required a mean of 20 min (95%CI 13,28).

#### Phlebotomy encounter

The average time required by phlebotomy staff for a blood draw was 3.6 min (95%CI 3.0,4.2). There was no difference in average times between sites.

#### Administration encounter

The average time taken by administrative staff to check patients into and out of the outpatient clinic was 2.1 min (95%CI 1.8,2.4). There was no difference in the time required between the two sites. An additional 14 min of administrative time per patient was included to account for the time required to source the patient medical records (4 min) and type the letter (10 min) to the patients’ general practitioner.

The overall cost of an outpatient HCV medical review including staff utilisation *(medical, administration, phlebotomy services)* (€41) and laboratory/radiology tests (€95) was estimated to be €136 (€3.60 SD).

The overall cost of a HCV clinical nurse specialist review including staff utilisation *(nursing, administration, phlebotomy services)* (€33) and laboratory/radiology investigations (€95) was estimated to be €128 (€7.30 SD).

### Annual healthcare resource utilisation of patients with hepatitis C

Two hundred and twenty-five patients were identified and categorised by their degree of liver disease, representing 819 patient-years of follow-up. One hundred and sixty-four (73 %) were male and the mean age of the cohort was 46 years. Table 2 presents a summary of the demographics and healthcare resource utilisation of these patients established through interrogation of the EPR.

**Table 2:** Summary of patient demographic and healthcare utilisation characteristics in differing HCV health-states

Characteristic	Mild	Mod.	Comp. Cirrhosis	Decomp. Cirrhosis	HCC	Transplant Year 1	Transplant > Year 1	SVR
N	40	29	24	13	27	33	31	28
Mean Age (SD)	38 (8)	48 (10)	45 (9)	46 (7)	55 (11)	47 (9)	54 (9)	34 (10)
Male n (%)	25 (61)	16 (55)	25 (89)	12 (98)	23 (85)	22 (67)	19 (66)	22 (79)
Patient years of follow-up	214	137	102	23	39	33	187	84
Annual mean number of medical reviews (95%CI)	1.3 (1.1, 1.5)	1.4 (1.2,1.7)	1.8 (1.5, 2.1)	4.6 (4.2, 4.9)	5.2 (4.6, 5.8)	11.9 (10.4, 13.2)	4.3 (4.0, 4.6)	0.36 (0.22, 0.5)
Annual mean number of nursing reviews (95 % CI)	0.5 (0.3, 0.7)	0.9 (0.6, 1.2)	1.8 (0.9, 2.6)	5.1 (4.3,5.9)	3.6 (3.1, 4.1)	n/a	n/a	0.1 (0.0, 0.04)
Annual mean number of inpatient episodes (95%CI)	n/a	n/a	0.2 (0.06, 0.4)	2.5 (2.1, 3.0)	2.1 (1.8, 2.3)	1.1 (0.6, 1.6)	0.24 (0.1, 0.38)	n/a

*Mod* moderate, *Comp. cirrhosis* Compensated cirrhosis, *Decomp. Cirrhosis* decompensated cirrhosis, *HCC* hepatocellular carcinoma, *Transplant Year 1* First 12 months of liver transplantation, *Transplantation > Year 1* Transplantation After First 12 months, *SVR* Sustained Viral Response

Seventeen percent of the evaluated cohort had mild disease (*n* = 40). They accessed an average of 1.5 ambulatory care visits per year. Seventy percent (*n* = 28) had undergone a liver biopsy. Twenty-nine patients (13 %) had moderate disease and an average of 2.5 ambulatory care episodes per annum. Eighteen patients (62 %) had had a liver biopsy. Twenty-four patients (11 %) had compensated cirrhosis, of whom fourteen had had a liver biopsy performed (58 %) and sixteen had had upper GI endoscopy (67 %). Nine patients with compensated cirrhosis required a liver-related inpatient hospital stay (38 %) and one patient was admitted to the intensive care unit on four occasions.

Six percent of the evaluated cohort (*n* = 13) had decompensated cirrhosis. Eleven (85 %) required inpatient admissions. Four patients died over the course of the follow-up (31 %). Three patients (23 %) required an Intensive care unit admission. Eleven patients (85 %) underwent upper GI endoscopy; the majority (*n* = 7) had one procedure (range 0–2).

Twelve percent of the cohort (*n* = 27) had HCC. Twenty-two patients (81 %) required inpatient admissions. Six patients (22 %) received treatment with sorafenib for an average duration of four months (95%CI 3.1,5.5). Eighteen (67 %) had a trans-arterial chemo-embolisation (TACE) procedure carried out. The majority had this performed once (*n* = 10) however one patient had 6 procedures (95 % CI 0.98, 1.4). Sixteen patients died over the course of the follow-up (59 %) consistent with the high mortality seen with this condition.

Ten percent of the cohort (*n* = 28) had been treated successfully for HCV. The cost of their follow-up care was evaluated. Forty-six percent (*n* = 13) did not access HCV review in the three years post-SVR. Five patients (18 %) had cirrhosis. The mean number of annual medical reviews was 0.36 (95CI 0.2, 0.5). The annual mean cost of care post SVR was €44 (95 % CI €16, €73).

### Healthcare resource utilisation of patients receiving liver transplantation

The cost associated with the pre-transplant workup including staff costs, laboratory and radiological investigations was €2828. The inputs considered as part of the pre-transplant work-up are displayed in the Additional file 1. The DRG cost of the liver transplantation procedure in 2011 was €124,425. The mean number of outpatient reviews in the first year post-transplant was 12 (95%CI 10.4,13.2) and four (95%CI 4.0,4.6) in the years thereafter. The cost of an ambulatory care review including monitoring of immunosuppression was €184.54 (€3.60 SD) Sixteen patients (48 %) required re-admission under the liver service in the first year post-transplant. The mean number of admissions was one (95%CI 0.6,1.6). Fifteen (48 %) patients required an inpatient stay under the liver service in the years following their first year post-transplant. Prednisolone was the most commonly prescribed immunosuppressive in the first year post-transplant (88 %), followed by tacrolimus (81 %) and mycophenolate mofetil (75 %). In subsequent years the rate of prednisolone prescribing fell (33 %) and tacrolimus was the most commonly prescribed immunosuppressant (66 %). The cost of immunosuppressive drugs was €3713 per person in the first year and €3667 per person per year thereafter. The cost of antimicrobial prophylaxis was €1081 in the first year post-transplantation based on three months therapy with co-trimoxazole and either valaciclovir (79 %) or valganciclovir (21 %).

### Direct medical cost of HCV care in Ireland

**Fig. 1:**
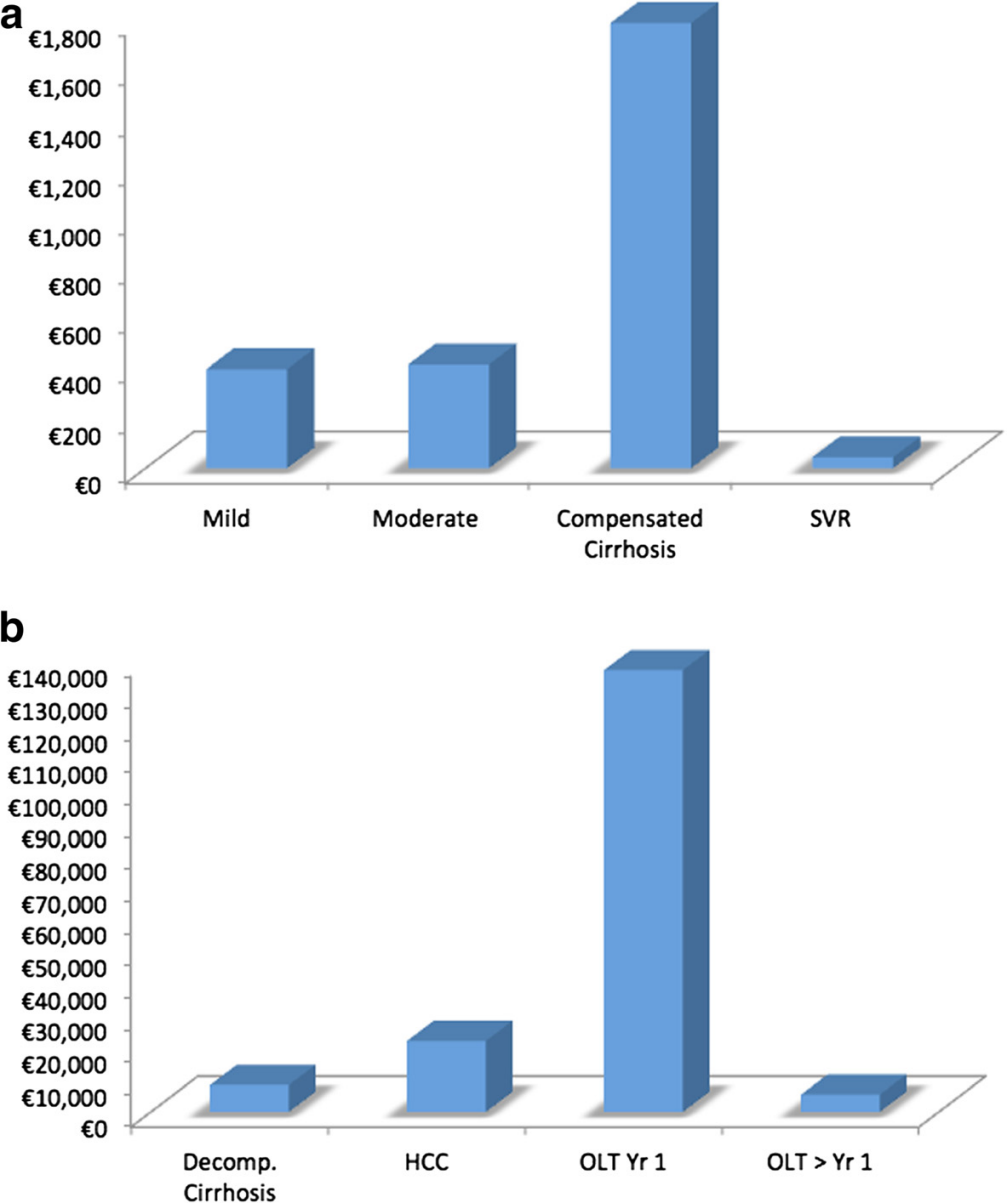
**a** Annual Direct Medical Cost of HCV care for milder HCV Health-states (y-axis in €100 s); **b** Annual Direct Medical Cost of HCV care for advanced HCV Health-states (y-axis in €10,000 s) *OLT = Orthotopic Liver Transplantation, HCC = Hepatocellular Carcinoma, Decomp. = Decompensated, SVR = Sustained Viral Response*

The direct medical costs of HCV care rose substantially with progression of liver disease (Fig. 1). Patients with mild liver disease accrued the least costs (€398) with patients in the first year post-transplantation accruing the most cost (€137,176). Patients with non-cirrhotic disease had significantly less cost than those with end-stage liver disease (ESLD). Table 3 summarises the mean cost and 95 % confidence intervals of the different HCV health-states.

**Table 3:** Annual mean direct medical costs of chronic HCV care for patient with different stages of liver disease in Ireland

HCV health-state	Mean annual cost	(95 % CI)
Mild	€398	(€336, €482)
Moderate	€417	(€335, €503)
Compensated cirrhosis	€1790	(€990, €3164)
Decompensated cirrhosis	€8303	(€3945, €14,637)
Hepatocellular carcinoma	€21,992	(€15,222, €29,467)
Transplant Year 1	€137,176	(€136,024, €138,306)
Transplant after Year 1	€5337	(€4942, €5799)
Sustained Virological Response	€44	(€16, €73)

## Discussion

The scale of the HCV epidemic and the rapidly expanding formulary of novel therapies undergoing regulatory assessment and re-imbursement decisions worldwide, mean that this disease area is of great interest in many healthcare systems [1, 16–19]. Treatment of HCV with pegylated interferon (PEG) and ribavirin has previously been shown to be cost–effective, as has triple therapy with PEG, ribavirin and a NS3/4A protease inhibitor [20–21]. However, uptake of these therapies was low due to their relatively limited effectiveness, long and complex regimens and substantial side-effects [22]. This resulted in a contained budget impact for payers although little advance from the perspective of patients with HCV. Future treatment options offer the possibility of all-oral treatment regimens with good tolerability and excellent efficacy [5–8]. As many of the previous barriers to HCV treatment have been comprehensively addressed, cost of treatment will become one of the most significant. Treatment costs may be offset by the reduction in the cost of chronic care elicited by successful treatment. However, real-world cost data for chronic HCV care is sparse internationally and not previously available in Ireland [16, 23–25]. Submissions to health-technology assessment agencies have been hampered by the lack of availability of good-quality, up-to-date cost estimates in this disease area with many of them utilising costs published in 2006 and established in 2003 [24, 26]. These are likely to be underestimates, particularly in the high-cost areas of hepatocellular carcinoma and liver transplantation, as treatment options such as sorafenib and TACE were not available in 2003 and prescribing practices in post-transplant immunosuppression have also changed [27]. This study demonstrates that the direct medical cost associated with HCV care in Ireland is substantial and that with progression of liver disease the costs associated with their care increase exponentially. This is consistent with recent reports from managed care insurance databases in the US, where the cost of HCV care increased substantially with the development of cirrhotic liver disease [16, 23, 28]. It is estimated that while the incidence of HCV is falling in many healthcare systems, the peak consumption of healthcare resources is yet to come as increasing numbers of patients with HCV develop complications of their chronic viral hepatitis [16, 29]. The cost of providing care to HCV patients will continue to rise unless treatment interventions are initiated to avert disease progression and avoid high-cost health-states. Given the large proportion of patients who are currently undiagnosed, treatment strategies aimed at future healthcare cost-containment will only be successful if patients are identified prior to their presentation with complications of end-stage liver disease. The importance of screening as part of this paradigm has been highlighted by recent international recommendations [3, 30]. The follow-up costs of patients who had achieved an SVR in this cohort were low in comparison to patients with ongoing chronic infection and significantly lower than those for patients with end-stage liver disease.

While the costs associated with advanced liver disease in HCV are substantial, the vast majority of patients with HCV at present have mild or moderate disease [23, 31]. The bulk of their management occurs through ambulatory care and therefore it is appropriate to develop accurate real-world unit costs of outpatient visits in this setting. Micro-costing is the recommended method of establishing unit costs although in practice it is rarely done due to time constraints [32, 33]. To our knowledge, this study presents the most detailed breakdown of a HCV routine outpatient visit. Cost studies have limitations when it comes to generalising outside of the health care system that they are derived in, as clinical practice and health care budgets vary internationally. Nonetheless, the detail provided will allow others to derive costs by applying unit costs reflective of their own practice to the resource utilisation of healthcare personnel and investigations presented, enhancing the generalisability and relevance of this data to those outside of Ireland. It provides a “bottom-up” perspective to HCV care that is lacking from cost estimates derived from large healthcare insurance databases and comprehensively establishes ambulatory and pharmacy direct cost estimates for all disease stages of HCV. The micro-costing study of ambulatory care was supplemented by DRG costs for inpatient admissions and procedures in order to give a more complete description of the cost of advanced HCV disease. DRG’s have limitations when used in economic modelling as they may not reflect the true cost of the admission and may not be representative of costs in other healthcare jurisdictions as they are derived from an individual country’s health care budget [34]. This is particularly relevant in the case of the liver transplantation Year 1 estimate, as the cost of the inpatient liver transplantation procedure predominates. Research in Ireland and in other countries has demonstrated that the DRG cost is most valid in disease areas not undergoing rapid technological or pharmacological changes [35, 36]. The inpatient management of advanced liver disease was felt to demonstrate these features and therefore DRG values were utilised. The unit DRG cost of liver transplantation, the component costs of post-transplant immunosuppression and prophylaxis and the number of post-transplant ambulatory care visits are stated so that international readers can assess how closely they reflect unit costs and clinical practice outside of Ireland.

### Limitations

This study does not consider costs associated with HCV treatment. The cost of HCV treatment in Ireland will be established through the prospectively collected HCV treatment registry established under the auspices of the Irish HCV Outcomes and Research Network (ICORN). This will provide real-world cost estimates of HCV therapy to inform cost-effectiveness and budget impact analysis in the future. As the study design was retrospective in nature it is possible that despite efforts to be as precise as possible with patient categorisation, some of the patients in the moderate and compensated cirrhosis groups may have transitioned to more advanced disease states over the course of the data capture. The numbers are likely to be small given the slow nature of disease progression in HCV and the relatively short time frame considered. Societal costs are not considered in this study as it concentrates on direct medical costs from the perspective of the payer. As histological criteria were used as part of the definition to establish the health-states that patients were placed in, a relatively large proportion of the cohort had received a liver biopsy. This may not reflect future practice with the increasing availability of transient elastometry. Cost studies have limitations when it comes to generalising outside of the health care system that they are derived in, as described above. A number of features peculiar to HCV care in Ireland merit special note: (1) the majority of HCV care in Ireland is provided by the publicly funded health care system and therefore these cost estimates may not reflect costs in healthcare systems with multiple payers (2) as treatment for drug dependency in Ireland is not linked with HCV services in Ireland (other than one small centre), the cost of drug dependency treatment was not considered, (3) the liver transplantation service in Ireland is not enrolled in Eurotransplant or other international organ matching procedures so costs associated with such procedures are not included in the analysis.

## Conclusion

HCV is an important public health area that is currently undergoing a tremendous expansion in therapeutic options that raise the possibility of effective cure for many patients. There will be challenges in many healthcare systems in funding these advances however and cost-effectiveness analysis will be important to establish the true value and opportunity cost associated with them. These detailed real world cost estimates will be of use to researchers, re-imbursement decision makers and clinicians alike as they consider the funding of the new direct acting anti-viral agents.

## Additional file

Additional file 1:
**Liver transplant work-up - staff utilisation and investigations.** (DOCX 86 kb)

## Abbreviations

HCV: Hepatitis C
HSE: Health service executive
SVR: Sustained virological response
DRG: Diagnosis Related Group
TACE: Trans-Arterial Chemo-embolisation
ANOVA: Analysis of variance
SPSS: Statistical package for the social sciences
95%CI: 95 % confidence interval
ESLD: End stage liver disease
PEG: Pegylated interferon
ICORN: Irish Hepatitis C Outcomes and Research Network
