# Supplementary material for: Hepatitis C in the era of direct-acting antivirals: real-world costs of untreated chronic hepatitis C; a cross-sectional study
Source: BMC Infect Dis. 2015 Oct 26;15:471. doi: 10.1186/s12879-015-1208-1 (PMC4624167; doi:10.1186/s12879-015-1208-1)
Supplement: Additional file 1: — Liver transplant work-up - staff utilisation and investigations. (DOCX 86 kb) [file 12879_2015_1208_MOESM1_ESM.docx]

| **Outpatient Reviews** | **UNITS** |
| --- | --- |
| Transplant co-ordinators | 120 |
| Anaesthetic review | 32 |
| Transplant surgical review 2-4 | 51 |
| Dietician review | 60 |
| Dental Review | 60 |
| Psychiatrist Review | 60 |
| Hepatology review | 20 |
| Laboratory |  |
| FBC | 1 |
| COAG | 1 |
| Group and Hold | 1 |
| Liver profile | 1 |
| Renal profile | 1 |
| Auto-antibodies | 1 |
| Immunoglobulins | 1 |
| AFP | 1 |
| alpha-1-antitrypsin +bone profile | 1 |
| Caeruloplasmin | 1 |
| Copper | 1 |
| HLA typing | 1 |
| Hepatitis A antibody | 1 |
| Hepatitis B Surface Antigen | 1 |
| Hepatitis C Antibody | 1 |
| Hepatitis C genotype | 1 |
| Hepatitis C viral load | 1 |
| CMV IgG | 1 |
| EBV IgG | 1 |
| HSV IgG | 1 |
| VZV IgG | 1 |
| Toxoplasma IgG | 1 |
| Adenovirus IgG | 1 |
| HIV Antibody | 1 |
| 24hour urine collection for protein and GFR | 2 |
|  |  |
| Radiology |  |
| CXR | 1 |
| OPG | 1 |
| CT abdomen | 1 |
| MRI liver (not standard) | 1 |
| Pelvic U/S (not standard) | 1 |
|  |  |
| Cardiac |  |
| ECG | 1 |
| ECHO | 1 |
| Right heart studies (<5%) | 0.1 |
|  |  |
| Respiratory |  |
| Pulmonary Function Tests | 1 |
| ABG | 1 |
|  |  |
